# Activation of c-Jun N-Terminal Kinase, a Potential Therapeutic Target in Autoimmune Arthritis

**DOI:** 10.3390/cells9112466

**Published:** 2020-11-12

**Authors:** Benjamin Lai, Chien-Hsiang Wu, Jenn-Haung Lai

**Affiliations:** 1 Center for Big Data Analytics and Statistics, Chang Gung Memorial Hospital, Linkou, Taoyuan 33305, Taiwan; benjaminlailai@gmail.com; 2 Division of Allergy, Immunology, and Rheumatology, Department of Internal Medicine, Chang Gung Memorial Hospital, Chang Gung University, Linkou, Taoyuan 33305, Taiwan; ali19900418@gmail.com; 3 Graduate Institute of Medical Science, National Defense Medical Center, Taipei 11490, Taiwan

**Keywords:** c-Jun N-terminal kinase, inflammation, autoimmune, arthritis

## Abstract

The c-Jun-N-terminal kinase (JNK) is a critical mediator involved in various physiological processes, such as immune responses, and the pathogenesis of various diseases, including autoimmune disorders. JNK is one of the crucial downstream signaling molecules of various immune triggers, mainly proinflammatory cytokines, in autoimmune arthritic conditions, mainly including rheumatoid arthritis, ankylosing spondylitis, and psoriatic arthritis. The activation of JNK is regulated in a complex manner by upstream kinases and phosphatases. Noticeably, different subtypes of JNKs behave differentially in immune responses. Furthermore, aside from biologics targeting proinflammatory cytokines, small-molecule inhibitors targeting signaling molecules such as Janus kinases can act as very powerful therapeutics in autoimmune arthritis patients unresponsiveness to conventional synthetic antirheumatic drugs. Nevertheless, despite these encouraging therapies, a population of patients with an inadequate therapeutic response to all currently available medications still remains. These findings identify the critical signaling molecule JNK as an attractive target for investigation of the immunopathogenesis of autoimmune disorders and for consideration as a potential therapeutic target for patients with autoimmune arthritis to achieve better disease control. This review provides a useful overview of the roles of JNK, how JNK is regulated in immunopathogenic responses, and the potential of therapeutically targeting JNK in patients with autoimmune arthritis.

## 1. Introduction

Autoimmune arthritis, which encompasses a group of diseases including mainly rheumatoid arthritis (RA), psoriatic arthritis (PsA), and ankylosing spondylitis (AS), contributes to the major factors resulting in joint and bone destruction. Patients with other autoimmune disorders, such as systemic lupus erythematosus (SLE) or inflammatory bowel disease (IBD), may also manifest with symptoms of arthritis. Examination of inflamed joints reveals extensive infiltration of activated immune effector cells, such as T lymphocytes, B lymphocytes, and macrophages. In addition, elevated levels of proinflammatory cytokines, such as tumor necrosis factor-alpha (TNF-α), interleukin (IL)-1, IL-6, and IL-17, secreted from these activated immune effector cells can be detected in serum, inflamed joint fluid, and soft tissues. These inflammatory cytokines can induce an autoregulatory circuit and further exaggerate inflammatory processes [1,2]. Under the effects of different cytokines, naïve T cells may differentiate toward T-helper (TH)-1, TH-2, TH-17, or regulatory T cells (Treg), which explains the complexity of immune responses. Accordingly, the intracellular signaling pathways that regulate the activation of immune cells and secretion of proinflammatory and anti-inflammatory cytokines are critical and affect the overall fate of immune responses [3,4,5].

Accumulated studies have identified several critical signaling pathways involved in the activation of the immune system leading to autoimmune arthritis. One of these is the mitogen-activated protein kinase (MAPK) signaling pathway. There are three well-recognized conventional MAPK pathways including the extracellular signal-regulated kinase (ERK), p38, and c-Jun N-terminal kinase (JNK) pathways that are critically involved in physiological processes, such as cell proliferation, cell differentiation, cell survival, cell death, and immune responses [6]. The activation of MAPK activity can also be readily detected in a variety of diseases, including diabetes, cancer, and autoimmunity disorders like autoimmune arthritis [7,8,9,10]. The signaling pathways regulating the activation of MAPK are generally evolutionarily conserved [7,11].

Stepwise therapeutic strategies have been suggested in the treatment guidelines for autoimmune arthritis and extensively applied to treat patients with autoimmune arthritis [12]. Initial treatment involves the prescription of conventional synthetic disease-modifying antirheumatic drugs (csDMARDs), such as methotrexate, hydroxychloroquine, cyclosporin, sulfasalazine, and leflunomide. If csDMARDs have inadequate therapeutic efficacy, biological DMARDs (bDMARDs) that specifically target proinflammatory cytokines, such as IL-6, IL-17, IL-23/IL-12, and TNF-α, or CD28 signaling will be applied [12,13]. In recent years, a major breakthrough in the development of orally delivered medications different from csDMARDs was the launch of small-molecule inhibitors specifically targeting signaling molecules, such as Janus kinases (JAKs), that regulate the critical signal transduction pathways of several proinflammatory cytokines to treat patients with autoimmune arthritis [12,14]. Despite all these encouraging developments, there is still a percentage of patients with autoimmune arthritis who do not respond well to currently available treatment options and still suffer from joint destruction.

The development of JAK inhibitors for the treatment of autoimmune arthritis suggests the possibility of targeting other signaling molecules, such as MAPK, to achieve better therapeutic benefits. Indeed, given this consideration, several small-molecule inhibitors targeting p38 have been developed and examined in clinical trials. Although blocking p38 showed some encouraging results in animal studies, this therapeutic approach is struggling in late-phase clinical trials because of the development of resistance or side effects [15,16,17]. The possibility of targeting another MAPK, JNK, has not been investigated in clinical trials. The potential of targeting JNK is supported by the recent observation that in patients who were initially diagnosed with undifferentiated arthritis, a population within this group developed RA after two years of follow-up, and these patients showed enhanced JNK activity compared to those who did not develop RA [18]. The study strongly suggests that JNK could be an attractive target for treatment of RA patients early in the disease process, a very challenging task even for experienced rheumatologists. In this review, we will go over JNK signaling and focus on (1) the evidence of JNK activation in inflamed joints, (2) how the activation of the JNK signaling pathway is immunologically triggered, (3) the pivotal upstream kinases and phosphatases regulating JNK activation, and (4) how different JNK subtypes may differentially contribute to the immunopathogenesis of joint inflammation. Finally, the possible benefit of developing JNK inhibitors for the treatment of autoimmune arthritis will be discussed.

## 2. Activation of JNK in Inflamed Joints

Increased phosphorylation of JNK can be readily detected in joint extracts from mice with collagen-induced arthritis (CIA) established by administering bovine type II collagen in complete Freund’s adjuvant, a commonly studied animal model simulating arthritis in patients with RA [19]. JNK activation can also be detected in joints in a mouse model established by injection of methylated bovine serum albumin in complete Freund’s adjuvant at the base of the tail [20] or in rats with adjuvant-induced arthritis (AIA) [21]. In humans, examination of synovial tissues has revealed evidence of activation of MAPKs, including ERK, JNK, and p38, in specimens from RA patients but not those from osteoarthritis (OA) patients. Interestingly, the locations of these three MAPKs appeared to be different. While activated ERK was localized around synovial microvessels and p38 was mainly in the synovial lining layer and synovial endothelial cells, activated JNK was localized around and within mononuclear cell infiltrates [22]. Activation of JNK could also be recognized in cells of the lining layer, in some of the sublining cell infiltrates, and in the perivascular compartment in synovial biopsies obtained from the knee joints of patients with psoriatic arthropathy [23]. JNK signaling is also critical in the pathogenesis of patients with ankylosing spondylitis [24]. These studies confirm the activated status of JNK in inflamed joints in different animal models of RA and patients with autoimmune arthritis. The significance of JNK activation in inflamed joints is reflected by studies showing that administration of the JNK-specific inhibitor SP600125 (anthra[1,9-cd]pyrazol-6(2H)-one) modestly decreases paw swelling and strikingly induces near-complete inhibition of radiographic joint damage, together with a reduction in the collagenase-3 level, in a rat model of AIA [21].

## 3. JNK Participates in Signaling by Critical Proinflammatory Cytokines in Autoimmune Arthritis

Various immune triggers are involved in the immunopathogenesis of autoimmune arthritis, among which the proinflammatory cytokines present at elevated levels in serum and inflamed joints play crucial roles [25]. Understanding how the JNK signaling pathway is involved in proinflammatory cytokine-mediated joint destruction in patients with autoimmune arthritis should be helpful in considering JNK pathway targeting as a therapeutic option to achieve better disease control. Currently, several cytokines, such as IL-1, IL-6, IL-17, IL-23/IL-12, and TNF-α, are the major targets of bDMARDs developed in past decades to treat autoimmune arthritis patients with an inadequate therapeutic response to csDMARDs [26]. Activation of JNK pathway by different cytokines can be detected in different tissue cells or animal models (discussed below). However, the direct roles of JNK in many cytokine-mediated effects have not yet been solidly demonstrated by approaches like siRNA introduction, specific chemical inhibitors, or knockout (KO) studies. Accordingly, instead of extensively discussing the regulation of JNK by these proinflammatory cytokines, we specifically emphasized the roles of JNK in TNF-α- and IL-17-mediated inflammatory responses in autoimmune arthritis. Being recognized as an anti-inflammatory cytokine, IL-4 effects on JNK were also briefly discussed.

### 3.1. TNF-α, Arthritis, and JNK

The involvement of TNF-α in the pathogenesis of arthritis was initially suggested by several in vitro approaches demonstrating that TNF-α can stimulate collagenases and many other matrix metalloproteinases (MMPs) in human synovial cells and fibroblasts [27,28] and recombinant TNF-α can cause bone resorption and inhibit bone formation [29]. Subsequent studies suggest that TNF-α may play an upstream role in the cytokine cascades occurring in several inflammatory reactions as blocking TNF-α bioactivity results in the inhibition of IL-1 production and neutralization of IL-1 bioactivity in synovial cells [30]. Furthermore, blockade of TNF-α also inhibits the production of several other proinflammatory or immunoregulatory cytokines, including granulocyte-macrophage colony-stimulating factor, IL-6 and IL-8 [31]. Examination of synovial specimens from inflamed joints in patients with AS, RA, or juvenile RA showed overexpression of TNF-α at the mRNA and protein levels and TNF-α receptor, and TNF-α-positive cells mainly colocalized with CD68+ macrophages [32,33,34,35]. In addition to macrophages, T cells infiltrating inflamed joints also contribute to the production of TNF-α [36]. A less-recognized population of effector immune cells, natural killer cells, can also produce a large amount of TNF-α and contribute to the immunopathogenesis of autoinflammatory diseases, such as systemic juvenile idiopathic arthritis [37].

Mice carrying a human TNF transgene develop chronic inflammatory polyarthritis, and the administration of an anti-TNF monoclonal antibody to these arthritic mice completely prevents disease development, suggesting a direct and critical role for TNF in the pathogenesis of polyarthritis [38]. Consistently, KO of TNF-like ligand 1A, which is a member of the TNF superfamily, also produces a decrease in clinical severity in mice with CIA, providing supporting evidence that TNF-α plays a role in autoimmune arthritis [39]. The importance of TNF-α was confirmed by experiments in which anti-TNF neutralizing antibodies were administered, which resulted in reductions in paw swelling, the histological severity of arthritis, and the clinical score for arthritis in a mouse model of CIA [40]. An early-phase clinical trial enrolling 20 patients revealed that anti-TNF treatment resulted in reductions in the swollen joint count and Ritchie Articular Index as well as decreases in C-reactive protein and IL-6 serum levels [41]. A randomized double-blind trial including 73 patients with active RA further confirmed the therapeutic efficacy of anti-TNF treatment by measuring both laboratory and clinical parameters for the disease as well as monitoring tolerable adverse events in treated patients [42]. The synergism between anti-TNF treatment and low-dose methotrexate was confirmed in subsequent clinical trials including RA patients [43]. Currently, several different anti-TNF regimens have been developed and are commercially used to treat patients with various autoimmune arthritis conditions, although the presence of comorbid diseases may affect the choice of regimen [13].

TNF-mediated effects are mediated through both TNF receptor I (TNFRI) and TNFRII. While TNF-TNFRI interaction-mediated signaling events are generally proinflammatory, treatment with the TNFRII-selective fusion protein EHD2-sc-mTNFR2, which specifically binds to and activates TNFRII but has no effect on TNFRI, induces expansion of both CD4+ and CD8+ regulatory T cells (Tregs) in vivo, results in anti-inflammatory responses, and reduces the severity of arthritis in a mouse model of CIA [44]. The anti-inflammatory effects of signaling through TNFRII were also demonstrated by administration of the TNFRII-specific agonist TNCscTNF80 to a mouse CIA model [45]. To reduce adverse events due to global inhibition of TNF-mediated effects, therapeutics based on specifically targeting TNFRI with antagonists or targeting TNFRII with agonists are currently in preclinical and early clinical stages of development [46].

**Figure 1:**
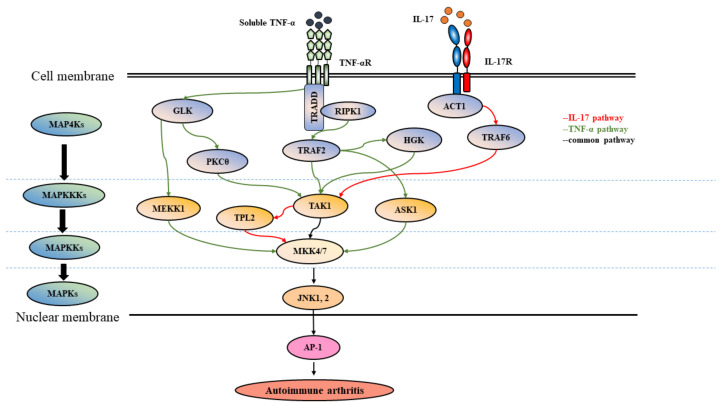
Activation of JNK by proinflammatory cytokines TNF-α and IL-17 leading to autoimmune arthritis. Binding of proinflammatory cytokine TNF-α or IL-17 to its receptor induces activation of JNK-AP-1 leading to autoimmune arthritis. Along the signaling pathways, many adaptors and kinases are involved. The sequences of activation are indicated with arrows. The TNF-α- and IL-17-mediated signaling pathways are shown in red color and green color, respectively. The common pathway shared by both TNF-α and IL-17 signals is illustrated in black color. Abbreviations: TNF-α, tumor necrosis factor-alpha; TNF-αR, TNF-α receptor; IL-17, interleukin-17; TNFR1, TNF receptor 1; TRADD, TNFR1-associated DD protein; RIPK1, receptor interacting serine/threonine-protein kinase 1; GCK, germinal center kinase; HPK, hematopoietic progenitor kinase; HGK, HPK1/GCK-like kinase; GLK, GCK-like kinase; ACT1, also known as CIKS (connection to inhibitor of κB (IκB) kinase and stress-activated protein kinases); HGK, HPK/GCK-like kinase; PKC-θ, protein kinase C-θ; TRAF2, TNFR-associated factor; TAK1, transforming growth factor-beta (TGF-β)-activated kinase 1; ASK1, apoptosis signal-regulating kinase 1; TPL2, tumor progression locus 2; MAPK4K, MAP kinase kinase kinase kinase; MAPK3K, MAP kinase kinase kinase; MAP2K, MAP kinase kinase; MAPK, mitogen-activated protein kinase; ERK, extracellular signal-regulated kinase; MEKK1, MAPK/ERK kinase kinase 1; MKK, mitogen-activated protein kinase kinase; AP-1, activator protein-1; JNK: c-Jun N-terminal Kinase.

TNF signaling through either TNFRI or TNFRII can activate MAPKs, including JNK. The interactions between TNF and its receptors initially induce the formation of a multiprotein signaling complex close to the cell membrane [47]. Given that many signaling events can be triggered by TNF stimulation, early studies have demonstrated that the activation of the JNK signaling pathway is required for TNF-α-mediated cellular apoptosis [48], a process involved in the development and pathogenesis of autoimmune arthritis [49,50]. Binding of TNFRI to TNF results in the recruitment of adapters such as TNFR1-associated DD protein (TRADD) and receptor interacting serine/threonine-protein kinase 1 (RIPK1) and further recruitment of TNFR-associated factor 2 (TRAF2), which induces formation of signaling complex I [51,52] (Figure 1). Activation of TRAF2 together with other molecules, such as E3 ubiquitin ligases, inhibitors of apoptosis protein (IAP)1/2 molecules, and transforming growth factor-beta (TGF-β)-activated kinase 1 (TAK1), leads to the activation of JNK [53,54,55]. In addition, the results of fusing the extracellular domain of the CD4 antigen to the intracellular domain of TNFRII and then providing stimulation with anti-CD4 antibodies suggest specific activation of JNK and ERK by TRFRII-mediated signaling [56]. Furthermore, dominant-negative TRAF2 that binds TNFRII blocks TNF-α-mediated JNK activation [56]. Additionally, the internalization and association of TNFRII with apoptosis signal-regulating kinase 1 (ASK1)-interacting protein-1 (AIP1)/ASK1 can also activate JNK [57].

Anti-TNF treatment with the biologic golimumab results in a reduction in immunological staining for JNK in synovial tissues from patients with RA [58]. Recently, Li et al., showed that constitutive low-intensity TNF stimulation but not short-term or high-intensity TNF stimulation induced persistent expression of osteoinductive Wnt proteins, which were expressed at significantly higher levels in the serum and spinal ligament tissues of AS patients, and led to bone formation. Treatment with chemical inhibitors or siRNA specific for JNK/AP-1 effectively blocked TNF-α-induced upregulation of Wnt expression [24]. The significance of the Wnt signaling pathway was further confirmed in a modified CIA model and a proteoglycan-induced spondylitis mouse model [24]. This report provided evidence suggesting that low-grade inflammation involving the TNF-JNK/c-Jun-Wnt signaling pathway may drive the new bone formation seen in patients with AS [24].

### 3.2. Interleukin-17 and JNK

The expression of IL-17 and IL-17 receptor is higher in synovial tissues from patients with RA or PsA than in those from patients with OA [59]. IL-17-positive cells are exclusively localized in the sublining layer of the synovium of patients with RA [60]. According to Cai et al., although IL-17 stimulation could upregulate the expression of a matrix metalloproteinase(s) in chondrocytes, the IL-17-mediated damage to the matrix appeared to be mainly induced through activation of an aggrecanase(s) but not a matrix metalloproteinase(s) [61]. However, studies also showed that injection of anti-rat IL-17A reduces arthritis index and MMP-13 level while it increases collagen type II alpha 1 expression in synovial or cartilage tissues in a rat CIA model [62]. The activation of JNK can be detected in human articular chondrocytes stimulated with IL-17 [63]. IL-17 triggers the immunopathogenesis of bony destruction in part by activating the JNK signaling pathway [64]. IL-17 stimulation also results in the induction of C-C motif chemokine ligand 2/monocyte chemoattractant protein 1, the monocyte chemoattractant mediating monocyte migration from the blood to synovial tissue, and the process involves the activation of the JNK pathway in RA synovial fibroblasts [65]. After stimulation with IL-17, the earliest event in signaling is the induction of the association between the IL-17 receptor and the adaptor ACT1, leading to activation of JNK [66]. Studies have shown that IL-17 enhances the expression of receptor activator of nuclear factor-kappaB (NF-κB) ligand (RANKL) and inhibits osteoprotegerin expression in human periodontal ligament cells, which exacerbates destructive processes in bone remodeling [67]. RANKL activates the TNF-receptor-associated factor (TRAF)-6 and JNK-c-Jun signaling pathways to mediate antiapoptotic effects on osteoclasts, and RANKL deprivation quickly causes osteoclast apoptosis [68]. In support of the importance of TRAF6 in IL-17-induced JNK activation, studies have shown that IL-17 fails to activate the JNK pathway in TRAF6-KO murine embryonic fibroblasts [69]. In bone marrow-derived macrophages, JNK1 enhances the degradation of TRAF3, an apoptosis regulator that modulates the antiapoptotic osteoclastogenic pathway and, therefore, promotes RANKL-induced osteoclastogenesis, and this effect is inhibited by treatment with the JNK-specific inhibitor SP600125 [70]. In a model of septic arthritis in mice, researchers showed that TH-17 cell-mediated osteoclast activation and bone resorption are associated with the activation of the NF-κB/JNK-RANKL axis [71]. Furthermore, IL-17A promotes osteoclast precursor autophagy and osteoclastogenesis at a low concentration by activating the RANKL-JNK pathway, and this effect is susceptible to suppression by treatment with the csDMARD chloroquine [72].

### 3.3. IL-4 and JNK

Several anti-inflammatory and immunoregulatory cytokines, such as IL-4, IL-13, IL-9, IL-33, IL-10, and IL-27, also play roles in autoimmune arthritis [73]. In this regard, the anti-inflammatory cytokine IL-4 may activate the JNK signaling pathway to induce a phenotypic shift in macrophages. Macrophages are separated into two major subpopulations that represent two different extreme states along a spectrum of activation statuses, the inflammatory M1 state and the regenerative M2 state [74]. In RA, the proportion of M1 macrophages exceeds that of M2 macrophages, and excessive activation is related to the increased inflammatory ability of macrophages, suggesting that the polarization of M1 and M2 macrophages is associated with autoimmune diseases [50]. In IL-4-activated macrophages, activation of macrophage scavenger receptor 1 (MSR1) enhances JNK activation and causes a phenotypic switch from the anti-inflammatory state to the proinflammatory state, and JNK inhibition reverses this effect [75]. Consistently, JNK inhibition can shift the polarization of proinflammatory M1 macrophages into the anti-inflammatory M2 state, causing a decrease in the expression of proinflammatory cytokines in obesity and insulin resistance animal models [76].

### 3.4. Upstream Kinases and Phosphatases Involved in Regulating JNK Activation in Autoimmune Arthritis

Many signaling molecules, such as kinases and phosphatases, and adaptor molecules, such as small GTP-binding proteins, are involved in connecting a signal at the cell surface to JNK. For generally recognized pathways of JNK activation, there are sequential signaling cascades from MAP kinase kinase kinase kinase (MAP4K) that activates MAP kinase kinase kinase (MAP3K), which stimulates MAP kinase kinase (MAP2K) by phosphorylating serine and/or threonine residues, and then MAP2K activates MAPK by dually phosphorylating a Thr-X-Tyr motif [77,78,79,80]. In addition to kinases and phosphatases, many posttranscriptional modifications, including acetylation, ubiquitination, and their reversals, are also tightly regulated in the activation of JNK [81]. While a full discussion of all these molecules and mechanisms is not possible, we focused on discussing some highly investigated upstream kinases/phosphatases, including MAP4K, MAP3K, and dual-specificity phosphatase (DUSP), in the activation of JNK leading to the pathogenesis of autoimmune arthritis.

### 3.5. MAP4K

Hematopoietic progenitor kinase 1 (HPK1), a hematopoietic cell-restricted member of the Ste20-related serine-threonine kinases, is widely expressed in effector immune cells, such as T cells, B cells, macrophages, and dendritic cells [82,83]. Studies showed that overexpression of HPK1 enhances activation of JNK stimulated with anti-CD3+anti-CD28 or phorbol 12-myristate 13-acetate (PMA)+ionomycin; however, ectopic expression of HPK1 inhibits T cell receptor (TCR)-mediated activation of AP-1 [84]. While cotransfection of expression vectors encoding HPK1 and JNK1 into Jurkat T cells causes enhanced phosphorylation of c-Jun, HPK1 deficiency results in enhanced TCR-mediated signaling events [84,85]. In addition, stimulation of TCR does not abolish JNK activation in primary T cells from HPK1-KO animals [85]. Further analysis revealed that HPK-1 inhibits anti-TCR- but not PMA-mediated activation of ERK2 [84], and yet, HPK1 deficiency leads to enhanced anti-CD3-mediated activation of ERK but not JNK or p38 [85]. These accumulated studies indicate that HPK activation solely depends on TCR; however, additional signal other than TCR for T cell activation may also activate JNK. Furthermore, these findings also suggest that in response to TCR stimulation, HPK1 may play dual roles in regulating the activation of JNK in T cells [80,84,85]. Bone marrow-derived DCs (BMDCs) from HPK1-KO mice exhibited a stronger capacity to stimulate T cell activation and proliferation than those from wild-type (WT) mice [86]. These results suggest that HPK1 acts as a negative regulator of DC functions. Interestingly, because HPK1 KO in murine primary B cells results in activation of JNK after stimulation through B cell receptor, HPK1 appears to play negative roles in B cell receptor-mediated activation and proliferation of B cells [87]. Examination of peripheral blood cells from patients with active psoriatic arthritis revealed reduced levels of HPK1 compared to those in peripheral blood cells from a control population [88]. In addition, both the mRNA and protein levels of HPK1 were significantly reduced in patients with SLE, which was not correlated with the prescribed medications for the patients [89]. Furthermore, a reduction in the HPK1 level appeared to show correlations with accelerated T cell proliferation and production of interferon-γ (IFN-γ) and IgG. Molecular analysis revealed that the inhibition of HPK1 expression in CD4+ T cells of patients with SLE might be due to the loss of jumonji domain-containing protein D3 binding and increased histone H3 lysine 27 trimethylation enrichment at the HPK1 promoter, resulting in the overactivation of T cells and B cells [89].

When transiently expressed in 293 cells, GCK-like kinase (GLK), one of the downstream signaling molecules in the TNF-α cascade, could specifically activate JNK but not ERK or p38 at least partly through the activation of mitogen-activated protein kinase/ERK kinase kinase-1 (MEKK1) [90]. Deletion of amino acids 353–835 in the putative C-terminal domain or mutation of Lys-35 in the putative ATP-binding domain significantly attenuated the ability of GLK to activate JNK [90]. Animals with GLK deficiency show defects in antibody production and reduced serum levels of TH-1/TH-2/TH-17 cytokines in response to immunization with a T cell-dependent antigen, keyhole limpet hemocyanin [91]. The GLK-mediated immunoregulatory effects were likely mediated through its direct interaction with and activation of protein kinase C-θ, leading to TAK1 activation [91]. An analysis of T cells from patients with SLE revealed a marked increase in the numbers of GLK+ T cells but not those of GLK+ B cells, and the frequency of GLK-expressing T cells correlated well with the measured SLE disease activity [91]. In patients with adult-onset Still’s disease (AOSD) who may present with polyarthritis, Chen et al., observed increased mRNA and protein expression of GLK compared to that in healthy controls, and the frequencies of GLK+ T cells were highly correlated with disease activity [92]. A correlation was also observed between the number of GLK+ T cells and the serum levels of IL-6 and IL-17 in these AOSD patients [92]. The same group of researchers further demonstrated protection from joint damage in GLK-deficient mice with CIA compared to WT mice. Moreover, increased expression of GLK was also observed in T cells in synovial fluid and synovial tissues from patients with RA compared to those with OA, and a significant correlation between the frequency of GLK+ T cells and RA disease activity was found [93].

Although not yet examined in a model of autoimmune arthritis, Map4K4 or Nck-interacting kinase (NIK), which was initially cloned from an adult mouse brain cDNA library and its human isolate designated HPK/GCK-like kinase (HGK), may also have potential, given its specificity in activating JNK but not p38 or ERK in the immune response [94,95]. Experiments revealed that dominant-negative HGK mutants inhibited TNF-α-induced JNK activation and that HGK-induced JNK activation was inhibited by dominant-negative MKK4 and MKK7 mutants [95]. Because mice with Nik deficiency die after gastrulation between embryonic days 9.5 and 10.5 [96], different approaches were used to investigate the roles of this molecule. Conditional deletion of HGK specifically in T cells resulted in severe dermatitis and cataracts as well as an autoinflammatory reaction manifesting with hepatosplenomegaly and lymph node enlargement in mice [97]. In addition, diffuse infiltration of immune cells could be detected in various organs and tissues, such as the skin, eyes, liver, and lungs. There were increased serum levels of IL-6 and IL-17 but not IFN-γ or TNF-α in mice with conditional deletion of HGK in T cells [97]. Studies revealed that HGK might directly phosphorylate TRAF2, leading to TRAF2 degradation in lysosomes [97]. Showing specificity, HGK only interacts with TRAF2, not other TRAFs, in resting T cells [97]. From these experiments, it seems reasonable to conclude that HGK is critical in maintaining T cells in a resting status and that in the absence of HGK in T cells, the basal level of TRAF2 increases, leading to increased production of several proinflammatory cytokines. The association between HGK and autoimmune arthritis may warrant investigation. An excellent review discussing the role of MAP4K in SLE has recently been published [98].

### 3.6. MAP3K

MAP3K tumor progression locus 2 (TPL2) regulates the activation of JNK and other MAPKs induced by various proinflammatory signals, such as IL-1β, TNF-α, and IL-17 [99,100,101,102]. The examination of skin lesions in patients with psoriasis and the joint capsule in patients with RA revealed high mRNA expression of TPL2 [103]. Administration of selective inhibitors of TPL2 significantly attenuated joint inflammation in rats with CIA, and the therapeutic benefits were also identified in examinations of joint cortical bone volume and a histological disease index [103], phenomena that were also reproducibly detected by examining the inflamed joints in mice with mannan-induced arthritis [103]. Noticeably, protection from bone erosion and cartilage damage in TPL2 inhibitor-treated animals were also comparable to those observed in animals treated with a TNF inhibitor [103]. The severity of psoriasis-like skin lesions on the feet, which were generated by intraperitoneal injection of mannan from *Saccharomyces cerevisiae* that elicited clinical features similar to those in patients with psoriasis and psoriatic arthritis, was also diminished in TPL2-KO mice compared to WT mice [103]. The therapeutic potential of inflammation blockade has also been demonstrated in other inflammatory disorders, such as IBD, that have the potential for patients to manifest with autoimmune arthritis [104,105]. The therapeutic benefits of TPL2 blockade in a psoriatic arthritis model can be explained by the involvement of TPL2 in the IL-17-mediated signaling pathway [99].

### 3.7. Phosphatases

In addition to the regulation mediated by kinases, dephosphorylation events also tightly regulate MAPK activation. In contrast to kinases, which often activate proteins, phosphatases dephosphorylate proteins, leading to the inhibition of protein effects and associated downstream signaling pathways. Accordingly, dephosphorylation of JNK is also a critical step in JNK activation regulation. Several DUSPs play critical roles in the immunopathogenesis of autoimmune arthritis.

The examination of synovial biopsies from patients with RA and OA revealed reduced DUSP1 expression, suggesting that DUSP1 contributes to protection from joint inflammation. Indeed, the DUSP1 deficiency in DUSP-1^−/−^ mice results in an increased severity of arthritis, higher numbers of osteoclasts in inflamed joints, and more extensive bone loss in a mouse model of CIA [106]. However, the protective role of DUSP1 in bone homeostasis in mice with strong inflammation is not reflected in contexts of limited inflammation, such as age-related spontaneously occurring OA [107]. Somewhat different from the effect of DUSP1 KO, aged mice with DUSP22 deficiency spontaneously develop inflammation and autoimmunity manifesting with elevated circulating levels of antinuclear antibodies and antidouble-stranded DNA antibodies, together with reductions in the levels of proinflammatory cytokines, such as IFN-γ, IL-17, IL-6, and TNF-α in the serum, compared with young DUSP22-KO mice [108]. Furthermore, several distinctive features, such as glomerular atrophy, mesangial hypertrophy, and mononuclear cell infiltration, are also more pronounced in aged DUSP22-KO mice than in young DUSP22-KO mice [108]. All these features tightly link the crucial roles of DUSP22 with autoimmune disorders, such as SLE [108].

## 4. JNK Subtypes Differentially Contribute to the Immunopathogenesis of Autoimmune Arthritis

Activation of the JNK pathway may lead to different effects according to the subtypes of JNK involved and the location and circumstances in which the enzyme is activated [109,110,111,112,113] (Table 1). There are three JNK subtype genes encoding different JNKs, namely, *jnk1*, *jnk2*, and *jnk3* [77]. Both JNK1 and JNK2 are ubiquitously expressed; however, the expression of JNK3 is limited to the brain, heart, and testis [77]. While simultaneous KO of JNK1 and JNK2 leads to embryonic lethality, mice with KO of JNK1, JNK2, or JNK3 are viable [114,115,116]. There is no upregulation of JNK1 expression in JNK2-deficient cells or of JNK2 expression in JNK1-deficient cells. In a study, mice with JNK2 deficiency (Jnk2(−/−) mice) appeared to develop slightly more severe arthritis symptoms than WT mice. However, there were no significant changes in histological scores for synovial inflammation between JNK2-KO mice and WT mice, but there was significantly less joint damage determined by safranin O-staining of cartilage in JNK2-KO mice than in WT mice. The study suggests that JNK-2 is more likely to affect the degradation of the matrix than to affect joint inflammation [117].

In the absence of JNK1, the capacity to generate IL-17+ T cells becomes defective, accompanied by enhanced IL-10 production and a defective response to infection by certain microbes as well as progression of neuroinflammation [118]. According to Guma et al., mice with JNK1 deficiency but not JNK2 deficiency showed significant reductions in inflammatory cell infiltration and joint damage in methylated bovine serum albumin-induced arthritis [20]. While the production of cytokines and chemokines by macrophages with JNK1 deficiency appeared to be unchanged compared to that by macrophages from WT mice, the migration of macrophages was impaired in the JNK1-KO context [20]. In addition, treatment with the peptide inhibitor D-JNKi dramatically reduced inflammation and joint destruction in WT mice with AIA [20]. In two experimental arthritis models, namely, CIA and KRN TCR-transgenic mice used to establish C57BL/6 nonobese diabetic serum transfer arthritis models, Denninger et al., demonstrated that JNK1 deficiency led to protection from arthritis [110]. In contrast, JNK2 deficiency worsened the symptoms of arthritis. The differential expression of CD86 on macrophages in JNK1-KO and JNK2-KO animals resulted in alterations in T cell immunity, which partly explains the different outcomes for arthritis protection [110]. Consistently, using a serum transfer model of arthritis, the development of arthritis and joint destruction was found to be dependent on JNK1 but not JNK2. Interestingly, the authors found that bone marrow-derived cells, especially mast cells, are responsible for the proinflammatory activity of JNK1, which regulates mast cell degranulation and Fcγ receptor (FcγR)-triggered IL-1β production [120]. Studies also revealed that an efficient step in osteoclastogenesis by bone marrow monocytes required the activation of JNK1 but not JNK2 and that JNK1 provided protective signals to prevent RANKL-induced apoptosis during bone marrow monocyte differentiation [119]. Altogether, although there are some not exactly consistent results in animal studies with KO of JNK1 or JNK2, most studies suggest that JNK1 appears to play more damaging roles than JNK2 in the pathogenesis of autoimmune arthritis.

While most studies suggest that JNK1 may be the primary subtype of JNK mediating the proinflammatory response and has potential as a therapeutic target in patients with autoimmune arthritis, some examples suggest different conclusions. In human TNF-transgenic mice that spontaneously develop inflammatory arthritis, the quantities of synovial inflammation and bone erosion were not significantly different between JNK1-deficient mice and WT mice. The expression of JNK2 and phosphorylation of c-Jun remain intact in mice with JNK1 deficiency, which suggests that JNK2 may compensate for JNK1 and that JNK1 may not be essential in TNF-mediated inflammatory arthritis [121]. In mice with age-related OA, deletion of JNK1 or JNK2 is associated with increased severity, suggesting that JNK may negatively affect senescence in the joints [122]. These results suggest that, in some circumstances, the JNK pathway may have an anti-inflammatory or antiaging effect.

## 5. JNK Inhibitors with Potential as Therapeutics in Autoimmune Arthritis

Several currently available csDMARDs and bDMARDs that preserve different immunomodulatory mechanisms in the treatment of autoimmune arthritis have been shown to inhibit JNK pathways, although this inhibition is generally not specific [58,123,124]. During the past few decades, scientists have tried to develop JNK-specific inhibitors for a variety of purposes. By analyzing the structures of JNK in complex with various inhibitors, approximately 100 JNK structures with various compounds have been identified, and the characteristics of these inhibitors, such as inhibitors of the open conformation or closed conformation of the gatekeeper residue, non-ATP site binders, covalent inhibitors, and type II kinase inhibitors, have been reported recently [125]. In addition, some compounds that specifically inhibit JNK activity were demonstrated to have the ability to attenuate arthritis in animal studies. For example, in a murine CIA model, IQ-1S administration either before or after arthritis induction produced decreases in inflammation and cartilage loss [126]. Docking of the IQ-1S syn isomer into the JNK1 binding site corresponds to the position of the cocrystallized JNK inhibitor SP600125, suggesting that IQ-1S is a high-affinity JNK inhibitor. As part of the protective mechanisms, IQ-1S also increased the number of regulatory T cells present in lymph nodes. The JNK-specific inhibitor CC-930 prevented dermal thickening, myofibroblast differentiation, and collagen accumulation in an animal model of systemic sclerosis, an autoimmune disorder that may also manifest with autoimmune arthritis [127]. In addition to JNK, other potential targets are JNK-interacting proteins (JIPs). There are four JIP subtypes [55]. Studies showed that CIA mice with JIP3 deficiency presented with significant decreases in an arthritis index and the swollen joint count compared to WT mice. In addition to the inhibition of the JNK/c-Jun pathway, the activation of the RANKL/RANK/OPG pathway was blocked in JIP3-KO mice [128]. Accordingly, pharmacological targeting of JIPs may also provide a therapeutic option for specific inhibition of JNK activity.

Recent accomplishments may also facilitate successful development of JNK-specific inhibitors. Mukaro et al., identified TNFRI sequence-specific TNFRI206-211 peptides that bound to TNF and inhibited TNF-induced p38 activation, respiratory burs, and cytokine production in neutrophils [129]. In vivo studies showed that TNFRI206-211 inhibited carrageenan-induced and antigen-induced paw inflammation in mice [129]. Specifically, TNFRI206-211 had no effect on the TNF-induced activation of ERK, JNK, and NF-κB. Likewise, a TNFR domain responsible for connecting TNF-JNK signaling may be identified and characterized, which would help in the design of specific inhibitors of JNK but not p38 or ERK to influence TNF-mediated effects. To date, combining both JNK and arthritis to perform searches in ClinicalTrials.gov has not revealed any hits in the field. Nevertheless, the accumulated experience discussed above provides good evidence to support testing of the efficacy of JNK inhibitors in patients with autoimmune arthritis.

## Figures and Tables

**Table 1:** Arthritis-associated effects mediated by JNK subtypes.

	JNK1	JNK2	JNK3	Reference
Organ location	Ubiquitously	Ubiquitously	Limited	[77]
			Ex. Brain, Heart, and Testis	
Knockout mice	Viable	Viable	Viable	[114,115,116]
	Double knockout leads to embryonic lethality			
IL-17+ T cell production	Defective	Normal	Normal	[118]
Inflammatory cell infiltration into joints	+	−	−	[20]
Macrophage migration	+	−	−	[20]
CD86 expression	+	−	−	[110]
Development of arthritis and joint destruction	+	−	−	[110]
Osteoclastogenesis	+	−	−	[119]
RANKL-induced apoptosis	Protection	−	−	[119]
in bone marrow monocyte differentiation				
Mast cell degranulation	+	−	−	[120]

+ stands for mediating the effect. − stands for mediating a negative effect, mediating no such effects or not yet examined.
